# The impact of *Rhodiola rosea* on biomarkers of diabetes, inflammation, and microbiota in a leptin receptor-knockout mouse model

**DOI:** 10.1038/s41598-022-14241-7

**Published:** 2022-06-22

**Authors:** Mahtab Jafari, Jasmin Grace Juanson Arabit, Robert Courville, Dara Kiani, John M. Chaston, Cindy Duy Nguyen, Nilamani Jena, Zhong-Ying Liu, Prasanthi Tata, Richard A. Van Etten

**Affiliations:** 1 Department of Pharmaceutical Sciences, University of California, Irvine, Irvine, CA USA; 2 Department of Microbiology and Immunology, University of Illinois at Chicago College of Medicine, Chicago, IL USA; 3 Department of Plant and Wildlife Sciences, Brigham Young University, Provo, UT USA; 4 Department of Medicine, University of California, Irvine, Irvine, CA USA

**Keywords:** Biomarkers, Biomarkers, Endocrinology, Metabolic disorders

## Abstract

Type 2 diabetes is the most prevalent endocrine disease in the world, and recently the gut microbiota have become a potential target for its management. Recent studies have illustrated that this disease may predispose individuals to certain microbiome compositions, and treatments like metformin have been shown to change gut microbiota and their associated metabolic pathways. However, given the limitations and side effects associated with pharmaceuticals currently being used for therapy of diabetes, there is a significant need for alternative treatments. In this study, we investigated the effects of a root extract from *Rhodiola rosea* in a Leptin receptor knockout (*db/db*) mouse model of type 2 diabetes. Our previous work showed that *Rhodiola rosea* had anti-inflammatory and gut microbiome-modulating properties, while extending lifespan in several animal models. In this study, treatment with *Rhodiola rosea* improved fasting blood glucose levels, altered the response to exogenous insulin, and decreased circulating lipopolysaccharide and hepatic C-reactive protein transcript levels. We hypothesize that these changes may in part reflect the modulation of the microbiota, resulting in improved gut barrier integrity and decreasing the translocation of inflammatory biomolecules into the bloodstream. These findings indicate that *Rhodiola rosea* is an attractive candidate for further research in the management of type 2 diabetes.

## Introduction

Type 2 diabetes (T2D) is a metabolic disease that currently ranks as one of the largest concerns of global public health, affecting an estimated 476 million people worldwide^1^. With both genetic and environmental factors contributing to this complex disease, diabetes is a leading cause of mortality in many countries and globally impacts life expectancy in both developed and developing nations^1,2^. One of the hallmarks of the disease is hyperglycemia resulting from defects in insulin secretion, insulin action, or both^3^. Of the three forms of clinical diabetes, the vast majority (about 90%) of patients have type 2 diabetes (T2D), which is characterized by insulin resistance^3^. Whereas there are many environmental and behavioral factors that modulate genetic susceptibility to this form of diabetes, the prevalence of T2D has risen steadily in recent decades^4^. Due to rising health costs associated with the increasing incidence and prevalence of diabetes worldwide^5,6^, identifying and evaluating safe and cost-effective therapeutic interventions in the management of T2D is of increasing importance. Current treatment algorithms for T2D include lifestyle changes, as well as oral and parenteral drugs^7^. However, many current treatments have significant limitations or side effects that can impact this large patient population. Although metformin is first-line treatment for T2D, it has side effects including nausea and diarrhea and is contraindicated in patients predisposed to lactic acidosis^7,8^. Insulin secretagogues such as sulfonylureas and meglitinides tend to lose their efficacy over time due to beta cell failure^9,10^, while alpha-glucosidase inhibitors, thiazolidinediones, and dipeptidyl peptidase-4 inhibitors have unique mechanisms of action, but adverse effects such as weight gain, heart failure, and gastrointestinal issues have led to poor compliance with therapy^11–14^. Thus, there is a need for new therapeutic interventions in T2D that are safe and effective.

There is significant evidence that inflammation plays a central role in the pathogenesis of T2D through two distinct pathways^15^. Obesity, specifically visceral adiposity, causes systemic inflammation through infiltration of adipose tissue by macrophages^16,17^ and production of pro-inflammatory cytokines such as TNF-α, IL-1β, IL-6, and CCL2^18,19^, which act as antagonists of insulin signaling^20^. Recent studies also suggest that differences in the human gut microbiome between normal and diabetic subjects^21^ are linked to systemic inflammation through altered gut integrity^22,23^, increased circulating gram-negative bacteria and endotoxin^22,24^, as well as lipopolysaccharide (LPS)-induced inflammatory cytokine secretion through TLR-4 signaling^24,25^. Evidence in humans also suggests that certain gut microbiome compositions may predispose individuals to conditions such as obesity and diabetes^26,27^. As crucial components that moderate host health and physiology, the gut microbiome can contribute to inflammation, alterations of intestinal linings, dyslipidemia, and a wide variety of other changes^23^. However, studies also indicate that modulation of the gut microbiome can decrease LPS-induced systemic inflammation in a mouse T2D model, thereby counteracting these changes^25^. Together, these observations suggest that targeting inflammation through the microbiome could be a novel approach to treating T2D^17,28^.

Plant-derived therapeutics such as cinnamon and curcumin extracts have been shown to have potential anti-diabetic properties, although their efficacy has not been evaluated in randomized clinical trials^29,30^. The adaptogenic plant *Rhodiola rosea* is used as a medicinal in traditional medical practices worldwide and has been shown to have anti-inflammatory and gut microbiome-modulating properties^31–33^. As an adaptogen, *R. rosea* represents an important category of pharmacological substances that are known to aid the body in resisting a wide variety of stressors (i.e. biological, chemical, physical, etc.) to maintain homeostasis and stabilize physiological processes that may be disrupted^32^. *Rhodiola rosea* extract appears to be safe in human studies^34–36^, which makes it an attractive candidate for the treatment of T2D. *Rhodiola rosea* extended lifespan in several animal models including worms, snails, and flies^37–40^. When tested on a fly model deficient in the insulin receptor substrate *chico*, *R. rosea* still extended lifespan but decreased expression levels of *Drosophila* insulin-like peptide (dILP) 2, 3, and 5 in wild-type flies^39^, suggesting complex effects on the insulin signaling pathway. However, the effects of this plant extract on an animal model of diabetes have not yet been evaluated.

Here, we investigated the effects of a root extract from *R. rosea* in a Leptin receptor knockout (*db/db*) mouse model of T2D. Leptin is a key adipokine responsible for maintenance of energy homeostasis and body mass, whereas mice lacking the Leptin receptor display hyperphagia and consequently develop obesity, visceral adiposity, hyperglycemia, and hyperinsulinemia^41–43^. In other mouse models, deficiencies in Leptin as well as beta-cell dysfunction have been linked to T2D, suggesting that this adipokine plays a key role in the pathogenesis of diabetes^44^. Like human diabetics, *db/db* mice show augmented expression of the differentiation marker Aldh1a3 and reduced nuclear expression of the transcription factor Nkxx 6.1, and exhibit hyperglycemia compared to age-matched congenic non-*db/db* mice^45^. These observations make the *db/db* mouse model suitable for the goals of this study.

## Results

### *Rhodiola rosea* alters glucose homeostasis in diabetic *db/db* mice

**Figure 1:**
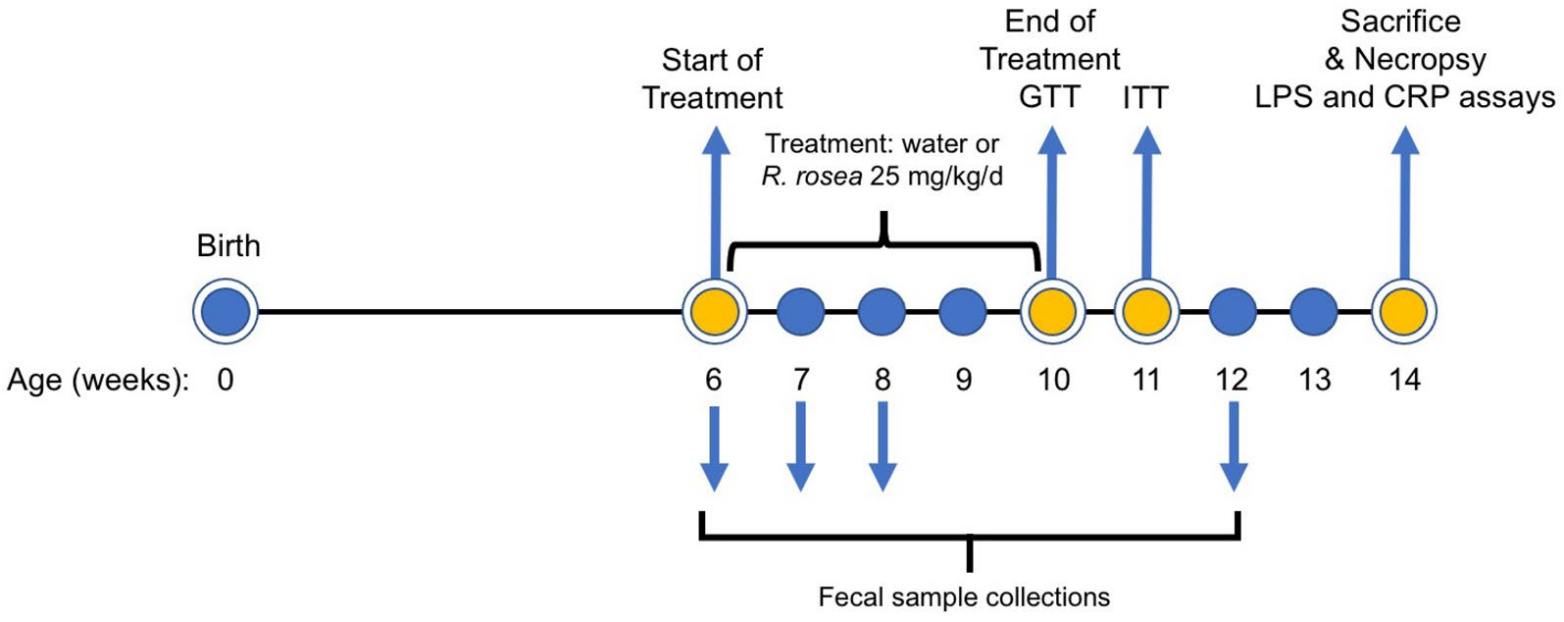
Timeline and experimental design. Cohorts of *db/db* mice were treated beginning at 6 weeks of age with *R. rosea* extract (25 mg/kg daily by oral gavage) or with water for a total of 4 weeks. A glucose tolerance test (GTT) was administered at the end of treatment (week 10), and an insulin tolerance test (ITT) administered 1 week later (week 11). Fecal samples were collected for microbiome analysis before treatment at week 6, and subsequently at weeks 7, 8 and 12.

**Figure 2:**
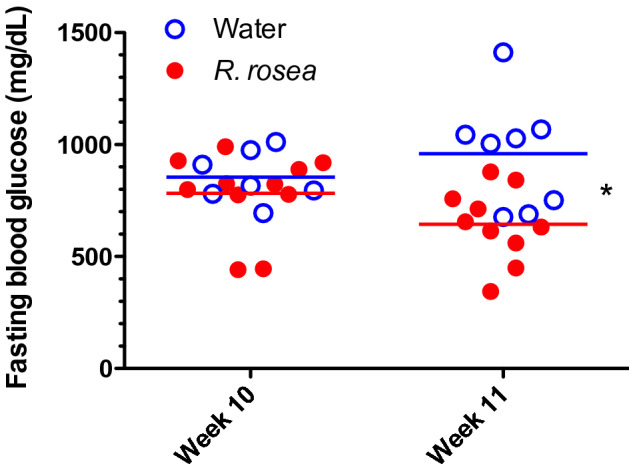
*Rhodiola rosea* improves fasting blood glucose in *db/db* mice. Folowing a 4 h fast, blood glucose levels were determined at the end of treatment (week 10) and 1 week later. The difference in mean blood glucose level at week 11 was significant (*P = 0.0303, repeated measures ANOVA). n = 8 control, n = 11 *R. rosea*-treated; one control sample from week 10 and one *R-rosea*-treated sample from week 11 did not give interpretable results and were omitted.

**Figure 3:**
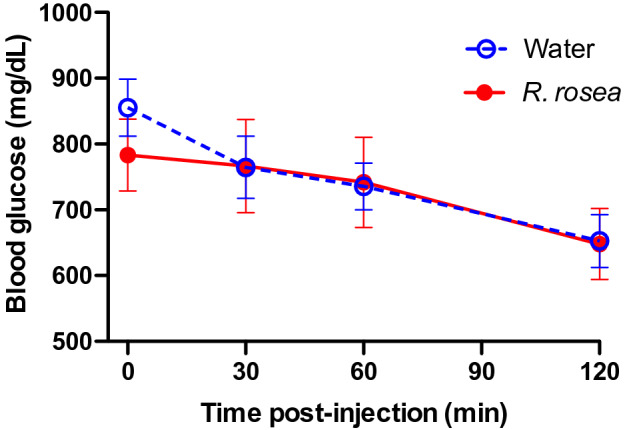
Lack of effect of *Rhodiola rosea* on glucose tolerance in *db/db* mice. Cohorts of mice (n = 7 control, n = 11 *R. rosea*-treated) at the end of the treatment period (week 10) were fasted for 4 h and then challenged with 0.5 g/kg glucose by intraperitoneal injection, followed by determination of blood glucose levels at 30, 60, and 120 min post-injection. None of the differences between control and *R. rosea*-treated mice were significant (one-way repeated measures ANOVA).

To assess the effects of *Rhodiola rosea* on a mouse model of T2D, we treated a cohort of 6 week-old leptin receptor-knockout (*db/db*) mice with an extract of *R. rosea* that was verified for quality based on validated biomarkers (see “Methods”), administered daily at a dose of 25 mg/kg by oral gavage for 4 weeks. The timeline of the study is depicted in Fig. 1. Control mice received gavage with water. We chose oral gavage as the route of admistration over adding the extract to food to allow more precise control of the amount of *R. rosea* administered. Following completion of treatment at 10 weeks of age, both *R. rosea*-treated and control *db/db* mice exhibited fasting hyperglycemia, with fasting blood glucose levels at 11 weeks of age significantly lower in *R. rosea*-treated mice than in control mice (Fig. 2). When subjected to a parenteral glucose challenge (glucose tolerance test, GTT) administered at the conclusion of treatment at week 10, there was no further elevation in blood glucose in either group, perhaps due to the extremely high fasting blood glucose levels in these mice, with a subsequent modest decrease in glucose levels over 120 min that might reflect induction of an endogenous insulin response (Fig. 3).

**Figure 4:**
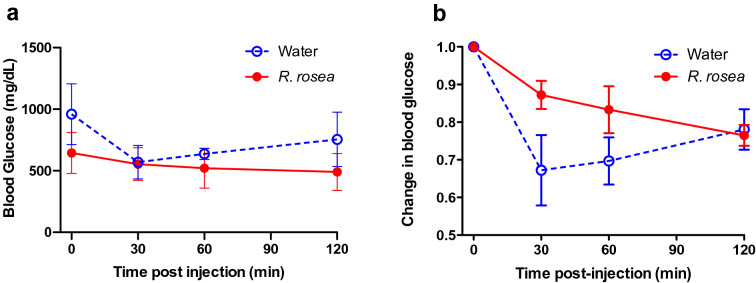
Altered insulin tolerance in *Rhodiola rosea*-treated *db/db* mice. Insulin tolerance test (ITT) of *db/db* mice. ITT was conducted at 11 weeks of age. Following a 4 h fast, mice were injected intraperitoneally with 0.75 IU/kg insulin. Blood was sampled pre-injection and at 30, 60 and 120 minues post-injection and glucose levels determined. The difference between the two data sets is significant (*P* < 0.05, one-way repeated measures ANOVA; n = 7 for water and n = 11 for *R. rosea*-treated).

Following a week of recovery, we tested the response of the two treatment cohorts to an exogenous insulin challenge (insulin tolerance test, ITT) at 11 weeks of age, 1 week after supplementation had ended (Fig. 4). *Rhodiola rosea*-treated mice showed a continuous decline in blood glucose levels over the 2 h period following insulin administration, with mean glucose values below those of water-treated mice at every time point (Fig. 4a). By contrast, the control mice exhibited an initial steeper decline in blood glucose in response to insulin (~ 33% decrease at 30 m; Fig. 4b) but a subsequent increase in blood glucose levels over the following 90 min. These results suggest that *R. rosea* treatment alters the response to insulin in diabetic *db/db* mice in a complex manner, resulting in a more prolonged response to insulin.

### Weight loss does not account for the effect of *Rhodiola rosea* on *db/db* mice

**Figure 5:**
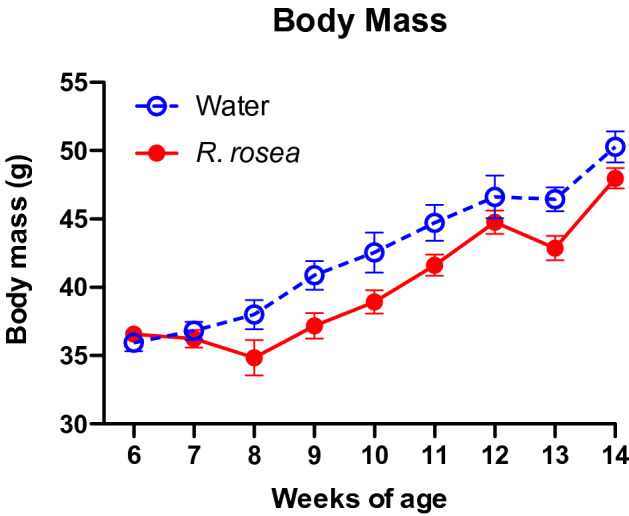
Effect of *Rhodiola rosea* treatment on body weight of *db/db* mice. Mice in each cohort (n = 8 water-treated, n = 11 *R. rosea*-treated) were weighed weekly starting just before initiation of treatment (week 6). None of the paired differences in weight were significant (one-way repeated measures ANOVA).

To assess whether changes in glucose homeostasis of *R. rosea-*supplemented mice were due to a decrease in obesity, we measured the body mass of the mice on a weekly basis during the study, beginning at the time of treatment initiation at week 6 (Fig. 5). The two cohorts had very similar mean weight before starting treatment, as expected. Treatment with *R. rosea* was associated with a transient decrease in weight, followed by a recovery in weight gain over time after week 8. Although the mean body weight of *R. rosea*-treated *db/db* mice was lower than control water-treated mice in weeks 8 through 14 of the study, these differences were not statistically significant.

### *Rhodiola rosea* modestly modulates the fecal microbiome of *db/db* mice

**Figure 6:**
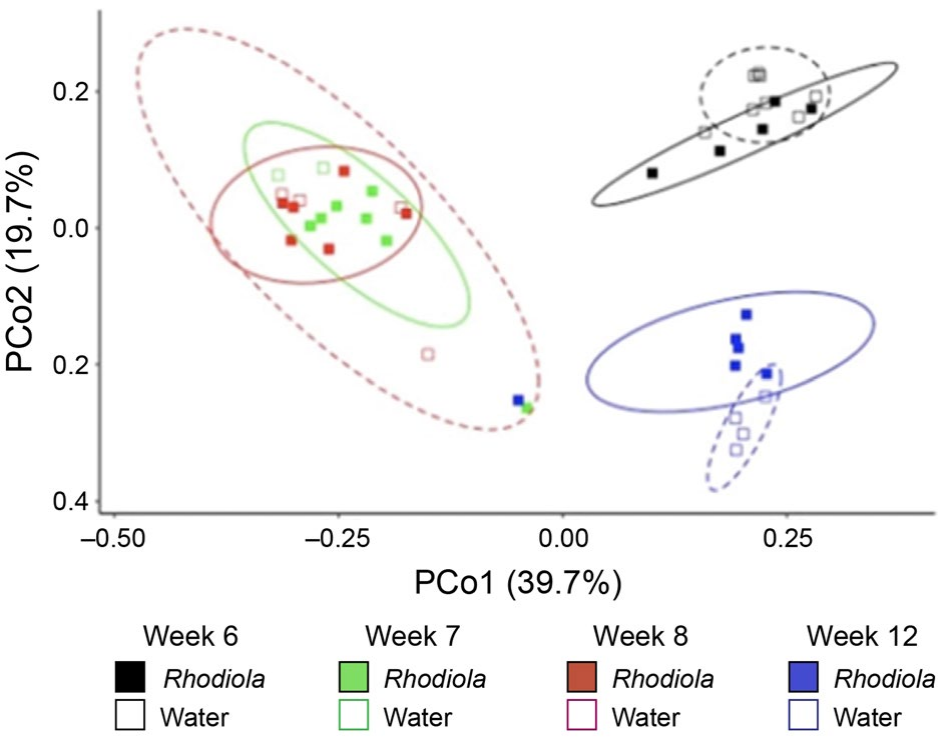
Effects of *Rhodiola rosea* on the fecal microbiome of *db/db* mice. Bray–Curtis principal coordinate ordination of the data from Supplemental Fig. 1. The corresponding PERMANOVA (Table 1) identifies significant differences in microbiota composition with treatment and time. The number of fecal samples from different mice analyzed is n = 5 (*R. rosea* week 6), n = 7 (*R. rosea* week 7), n = 6 (*R. rosea* week 8), n = 6 (*R. rosea* week 12), n = 8 (water week 6), n = 2 (water week 7), n = 4 (water week 8), and n = 4 (water week 12).

To assess the effect of *R. rosea* on the fecal microbiome of *db/db* mice, we collected serial stool samples before treatment (week 6) and at weeks 7, 8 and 12 (Fig. 1) from females of the two cohorts and analyzed the composition of the microbial community of the feces with 16S rRNA amplicon next-generation sequencing. The microbiome of both cohorts were dominated by *Bacteroidetes* and *Firmicutes*, and most of the reads could be designated as amplicon sequence variants (ASVs) within four major taxonomic assignments: the families *Rikenellaceae* and *S24-7* (*Bacteroidetes*), and the family *Lachnospiraceae* and genus *Lactobacillus* (*Firmicutes*). Compared to the treatment, time of sample collection accounted for the most variation in the microbiota composition of water and *R. rosea*-treated mice (Supplementary Fig. 1). When the microbiota composition of the two cohorts was subjected to Bray–Curtis analysis^46^, which does not account for phylogenetic relationships, there was a significant effect of *R. rosea* treatment on the fecal microbiome of *db/db* mice (Fig. 6 and Table 1). The only taxa that varied with time and treatment were reads assigned to the *Desulfovibrionales,* clustered at the order level, which were more abundant in *R. rosea*-treated mice than control mice (Supplementary Fig. 2). While a previous study demonstrated reduction in *Desulfovibrionales* abundance in the intestinal microbiota of BALB/c mice following treatment with salidroside^47^, a glycoside found in *R. rosea*, the difference in *Desulfovibrionales* abundance was observed before *R. rosea* was administered, suggesting the changes might be attributed to differences in microbiota composition before the experiment began (e.g. early cage effects) rather than to *R. rosea* treatment. Together, these findings identify modest but significant variation in the composition of the mouse fecal microbiota associated with *R. rosea* treatment.

**Table 1:** Results of PERMANOVA on Bray–Curtis distances of microbiota data.

	Degrees of freedom	Sum of squares	Mean squares	f	R^2^	*P* value
Treatment	1	0.2	0.2	3.64	0.04	0.02
Time point	3	2.89	0.96	17.69	0.55	0
Interaction	3	0.35	0.12	2.14	0.07	0.02
Residuals	34	1.85	0.05	NA	0.35	NA
Total	41	5.29	NA	NA	1	NA

### *Rhodiola rosea* decreases circulating lipopolysaccharide levels and a marker of inflammation

**Figure 7:**
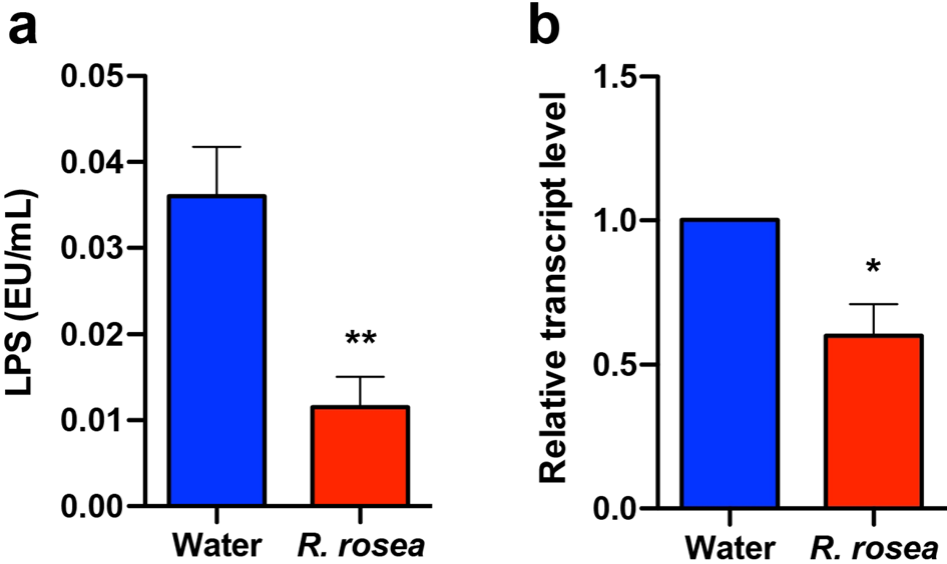
*Rhodiola rosea* treatment reduces circulating LPS and hepatocyte *CRP* expression. At 14 weeks of age, mice were sacrificed and serum and liver tissue obtained. (**a**) Serum LPS levels, measured by the LAL assay (n = 8 for water-treated and n = 6 for *R. rosea*-treated mice). **P < 0.01, Mann–Whitney U test. (**b**) Relative expression of *CRP* transcripts in mouse liver, calculated by the ΔΔCt method (n = 6 for water-treated and n = 5 for *R. rosea*-treated mice). *P < *0.05*, one sample Wilcoxon test.

To determine whether the changes of the fecal microbiota associated with *R. rosea* treatment had any physiological consequences, we determined the lipopolysaccharide (LPS) content from serum samples from the two cohorts via a limulus amoebocyte lysate (LAL) assay. Treatment with *R. rosea* decreased the LPS levels in the serum by almost 50% (Fig. 7a). Whereas circulating LPS (derived from gram-negative bacteria) triggers production of inflammatory cytokines by tissue macrophages and other cells, to further observe inflammation levels exhibited by the *db/db* mice, we determined if *R. rosea* treatment was associated with changes in C-reactive protein (CRP), an inflammatory marker exclusively produced by hepatocytes. Similar to LPS, *R. rosea* treatment decreased hepatic *CRP* transcript levels by about 40% (Fig. 7b).

## Discussion

The goal of this study was to evaluate the impact of *R. rosea*, a medicinal plant of emerging interest and possible therapeutic value, on the phenotype and the fecal microbiota of the *db/db* mouse model of T2D. Although this study shares a common format with a number of published microbiota-disease interaction articles^48–51^, it is one of the few studies that evaluates the impact of a botanical extract on the fecal microbiota throughout the progression of T2D. The format of our study was correlative and associative but the results may serve as the basis for future mechanism-based studies. We elected to use *db/db* mice for this study to test whether *R. rosea* can improve glucose homeostasis in a T2D model that is similar to severe and advanced human T2D in terms of visceral adiposity and insulin resistance, without resorting to a high-fat diet or other dietary manipulations. The use of this severe model may have prevented us from observing beneficial effects of *R. rosea* that might be relevant to less severe phenotypes of diabetes (i.e., pre-diabetes).

Daily treatment with *R. rosea* for 1 month significantly lowered the fasting blood glucose level in *db/db* mice at 11 weeks of age (Fig. 2). There was no effect of *R. rosea* on the response to a parenteral glucose load (Fig. 3), which may be a reflection of the substantial baseline hyperglycemia observed in both cohorts (Fig. 3 and reference 45). By contrast, *R. rosea* treatment significantly altered the response of *db/db* mice to exogenous insulin in a complex fashion, manifested as a decrease in acute blood glucose lowering but a sustained hypoglycemic response that persisted over 2 h (Fig. 4). These effects were not a consequence of decreased food intake and reduction in obesity (Fig. 5). Taken together, these results suggest that administration of *R. rosea* may favorably modulate the T2D phenotype, either by improving the function of insulin-responsive tissues in *db/db* mice or ameliorating the exhaustion of pancreatic beta cells that is observed in this model^45^.

Multiple studies have demonstrated major changes in the gut microbiota during the development of obesity^52^ and T2D in humans, including significantly reduced proportions of phylum *Firmicutes* and class *Clostridia*, in addition to compositional changes in the microbiota^27,53,54^. In addition, the presence of sulfate-reducing bacterial species from the *Desulfovibrionales* order has been associated with the pathology of T2D^21^. *db/db* mice display elevated levels of gut bacteria from the S24-7 family compared to wild-type mice^55^ and we observed a similar trend, with S24-7 bacteria dominating the composition of the fecal microbiome in both cohorts at all time points (Supplementary Fig. 1). While Bray–Curtis analysis demonstrated significant effects of *R. rosea* treatment on the *db/db* fecal microbiome (Fig. 6), our previous studies suggest that *R. rosea* does not modulate the gut microbiome through direct anti-microbial activity, as the plant extract did not suppress growth when tested on bacteria isolated from the *Drosophila* gut^33^.

As a consequence of changes in the local microbiome, gut integrity and intestinal permeability may be lowered through multiple mechanisms^19,22,25^, leading to endotoxemia and chronic inflammation^16^ that is postulated to contribute to the pathogenesis of T2D^16–19,26,27^. Mice with obesity induced by diet^25^ or genetic mutation (*db/db*)^56^ also have impaired gut integrity and increases in circulating LPS and inflammatory cytokines. Importantly, we observed significantly decreased levels of circulating LPS and decreased hepatic CRP transcripts (Fig. 7) in the *R. rosea*-treated cohort, providing a potential functional connection between modulation of the gut microbiome, inflammation, and glucose homeostasis by *R. rosea*. It is noteworthy that the changes in these inflammatory biomarkers persisted a month following cessation of *R. rosea* treatment, suggesting a long-term effect of exposure to *Rhodiola* on the pathophysiology of T2D in *db/db* mice.

In conclusion, while previous studies have illustrated that *R. rosea* has anti-inflammatory and gut microbiome-modulating properties and can extend lifespan in several animal models, the present study demonstrates that short-term exposure to *R. rosea* has beneficial effects on glucose homeostasis in the Leptin receptor knockout (*db/db*) mouse model of severe T2D, and suggests a possible mechanism of action. While only a modest modulation of the fecal microbiome was observed in this study, we hypothesize that these changes may have improved the integrity of the gut barrier, leading to decreased systemic inflammation. *Rhodiola rosea* is a good candidate for further investigation as a potential treatment for T2D, but further mechanistic studies in mice, and ultimately human clinical trials, are indicated.

## Methods

All methods were performed in accordance with applicable guidelines and regulations.

### Mouse strains and handling

BKS.Cg-*Dock7*^*m*+/+^ *Lepr*^*db*^/J and C57BL/6J mice were obtained from the Jackson Laboratory (Bar Harbor, ME). *Lepr*^*db*^/J mice were bred to generate homozygous *Lepr*^*db*^*/Lepr*^*db*^ (*db/db)* pups which were used to model T2D. During the study, mice were group-housed by sex in microisolator cages with filter tops (Techniplast) on a ventilated rack, provided with contact wood chip bedding (autoclaved Envigo Teklad corncob, 1/8 in.) and were allowed ad libitum access to food (Purina rodent chow #5001) and RO water. The animal room was maintained in 12 h light/dark cycles (0630 on/1830 off) at a temperature of 72° F ± 2° and humidity of 50% ± 5%. Enrichment was provided as two Nestlets (6 g, from Ancare) per cage. The animal facility was a SPF barrier facility; the health of the mice was monitored twice weekly by staff, testing for pathogens was done by monitoring exhaust dust and sentinels exposed to dirty bedding. Surveillance pathogen testing included Sendai virus, MHV, MPV, PVM, Reo-3, Mycoplasma, TMEV, LCMV, MVM, MNV, MKPV, EDIM, Ectromelia, pinworms, fur mites, *Helicobacter*, *C. bovis*, and *S. muris*.

Mice were weaned when they were 3 weeks old, and entered into the study at 6 weeks of age. At that point, mice were weighed, and mice of the same size (36 ± 1.5 g) randomly assigned to control (water) or experimental (*R. rosea*) treatment, with equal numbers of males and females per group. A cohort size of n = 9 was predicted to give 90% power to detect a decrease in fasting blood glucose from 800 to 600 mg/dL given an estimated standard deviation of 150 at a significance of α = 0.05 (one-sample *t*-test). At the end of the study, mice were humanely euthanized by a AVMA-approved method (CO_2_ asphyxiation using a gradual-fill method followed by cervical dislocation). The study was approved by the Instituitional Animal Care and Use Committee (IACUC) of UCI (Protocol #AUP-16-52). All experiments were performed in accordance with guidelines from UCI IACUC, U.S. Dept. of Agriculture APHIS, and ARRIVE version 2.0 (https://arriveguidelines.org).

### Study timeline

The study timeline is summarized in Fig. 1. Mice were administered either water (control) or 25 mg/kg *R. rosea* extract (treatment) by oral gavage daily for the 4 weeks of treatment, initiated when the mice were 6 weeks old and continued until the mice were 10 weeks old. This dose was selected based on our own preliminary dose-finding work and previously published studies where *R. rosea* was evaluated in rodent diabetes models^57,58^. The quality of the *R. rosea* extract was verified by HPLC that showed the extract contained 1.3% salidroside and 3.9% rosavins, consistent with a high-quality extract (data on file). Fecal samples were collected at 6 weeks of age (prior to treatment), 7 weeks of age, 8 weeks of age and 12 weeks of age. The glucose tolerance test (GTT) was performed at week 10 and insulin tolerance test (ITT) was performed at week 11. Mice were sacrificed and subjected to necropsy at week 14.

### Glucose tolerance test (GTT) and insulin tolerance test (ITT)

Given the importance of in vivo mouse models for studying the pathogenesis of T2D and various treatment interventions, several methods have been developed to investigate glucose tolerance, as well as the secretion and action of insulin in these models^59^. To ascertain potential differences between treatment and control groups with regards to glucose homeostasis, both glucose and insulin tolerance tests were performed. Four hours before the assays were performed, the food was removed to induce a state of fasting. Mice were placed briefly in an immobilization device without anesthesia, and blood samples (~ 2 µL) obtained from the mouse tail vein punctured by a 21 Ga needle. The first drop of blood was wiped away, and the second drop used to record values. Samples were blinded before determination of blood glucose levels using an AlphaTrak2 Blood Glucose Monitoring System. Blood glucose values were recorded 30, 60, and 120 min after intraperitoneal injection of either 0.5 g/kg glucose (5 μL per gram body weight of a 10% solution) or 0.75 IU/kg (3 μL per gram body weight of 0.25 IU/mL insulin stock). The number of mice utilized was 11 for the *R. rosea*-treated cohort and 7 water-treated control mice. Data were plotted using GraphPad Prism v8, statistical analysis was done in SAS using one-way repeated measures ANOVA.

### Body mass assay

Mice from both treatment cohorts (n = 8 for water-treated, n = 11 for *R. rosea*-treated) were weighed weekly from weeks 6–14. Data from male and female mice in each cohort were pooled and plotted using GraphPad Prism v8. Statistical analysis was performed in SAS using one-way repeated measures ANOVA.

### Microbiome analysis

Fecal samples from female mice were collected at 6 weeks of age (prior to treatment), 7 weeks of age, 8 weeks of age, and 12 weeks of age (Fig. 1). DNA was extracted using the Zymobiomics Mini DNA kit. 16S rRNA amplicon PCR was performed, targeting the V4-V5region using the EMP primers 515F (barcoded) and 926R^60^. The samples were prepared into a library that was sequenced at the UC Irvine Genomics High Throughput Facility on an Illumina MiSeq, using paired-end 300 bp v3 sequencing chemistry. The raw sequence data were imported into QIIME2 (qiime2.org)^61,62^ and demultiplexed^60^. This bioinformatics platform has recently been rewritten and reengineered for the next generation of microbiome sequencing, facilitating taxanomic and phylogenetic analyses. While dozens of software packages written in various programming languages are often needed for comprehensive analyses of this type of sequencing data, QIIME2 allows for “sequence quality checking, denoising, taxonomic classification, alignment, and phylogenetic tree building”—allowing for seamless analysis, description, and quantification of microbial communities^60^.

The sequences were assigned a taxonomic classification using the q2-feature-classifier^63^ on a GreenGenes database downloaded in July 2020 from the QIIME2 website (qiime2.org)^64^. A total of 6,352,455 read pairs passed Illumina quality filters. However, the quality scores of the reverse reads were generally lower than the forward reads, and no read pairs passed DADA2 quality filter steps^65^. Therefore, we analyzed the data only using the forward reads, which had higher average quality scores than the reverse reads. Forward reads that passed quality filtering and denoising by default DADA2 parameters in QIIME2 were rarefied to 11,390 reads per sample and beta-diversity distance metrics were calculated using QIIME2^46,66,67^ (Supplementary Fig. 3). As part of calculating the Unifrac beta-diversity metrics we constructed a phylogenetic tree with fasttree2^68^ based on mafft alignment^69^. Permutational multivariate analysis of variance (PERMANOVA)^70^ and analysis of microbial communities (ANCOM)^71^ analyses were performed in R.

### Limulus amebocyte lysate assay of serum LPS

Serum samples were obtained at week 14 (Fig. 1) from n = 6 *R. rosea*-treated mice and n = 9 control water-treated mice. Samples were blinded, diluted 40-fold, and LPS levels determined using the Pierce™ Chromogenic Endotoxin Quant Kit according to the manufacturer’s recommended protocol. Data were plotted and analyzed using GraphPad Prism v8.

### CRP transcript assay

Mice were sacrificed at week 14 (Fig. 1) and RNA extracted from liver tissue using Trizol. A total of 5 samples were collected from *R. rosea*-treated mice and 6 samples from control water-treated mice. Samples were blinded and the RNA extract was treated with DNA-free™ DNA Removal Kit from Invitrogen to remove any contaminating DNA. A cDNA library was generated with the iScript™ cDNA Synthesis Kit from Bio-Rad. qPCR was performed on a BioRadMJ Mini Personal Thermal Cycler with iQ SYBR Green Supermix. The PCR amplification program consisted of an initial denaturation set at 94 °C for 3 min, followed by 40 three-step cycles at 94 °C for 10 s, 60 °C at 30 s and at 72 °C for 45 s. The ΔΔCt Method was used to quantify the relative expression of genes of interest^46^. The *CRP* (gene of interest) and *GAPDH* (reference gene) primers were derived from PrimerBank (Supplementary Table 1). Data were plotted and analyzed using GraphPad Prism v8.

### Ethical statement

All experiments involving laboratory mice were carried out in compliance with ARRIVE guidelines 2.0 and with the approval of the UCI Institutional Animal Care and Use Committee (UCI IACUC; Animal Welfare Assurance # A3416-01) under the auspices of protocol AUP-16-52 (Van Etten laboratory; approval date 13 December 2016).

## Supplementary Information

Supplementary Information.

## Data Availability

16S rRNA metagenomic data are deposited in the NCBI Sequence Read Archive (https://www.ncbi.nlm.nih.gov/sra), accession # PRJNA848938. *CRP* transcript data are deposited in DRYAD (https://datadryad.org), 10.7280/D1FX2D. Other data in this manuscript are freely available to qualified researchers by contacting the lead author at mjafari@hs.uci.edu.
