# Supplementary material for: The impact of Rhodiola rosea on biomarkers of diabetes, inflammation, and microbiota in a leptin receptor-knockout mouse model
Source: Sci Rep. 2022 Jun 22;12:10581. doi: 10.1038/s41598-022-14241-7 (PMC9217815; doi:10.1038/s41598-022-14241-7)
Supplement: Supplementary file 1 — Supplementary Information. [file 41598_2022_14241_MOESM1_ESM.docx]

**SUPPLEMENTARY INFORMATION**

Jafari et al., The impact of *Rhodiola rosea* on biomarkers of diabetes, inflammation, and microbiota in a leptin receptor-knockout mouse model


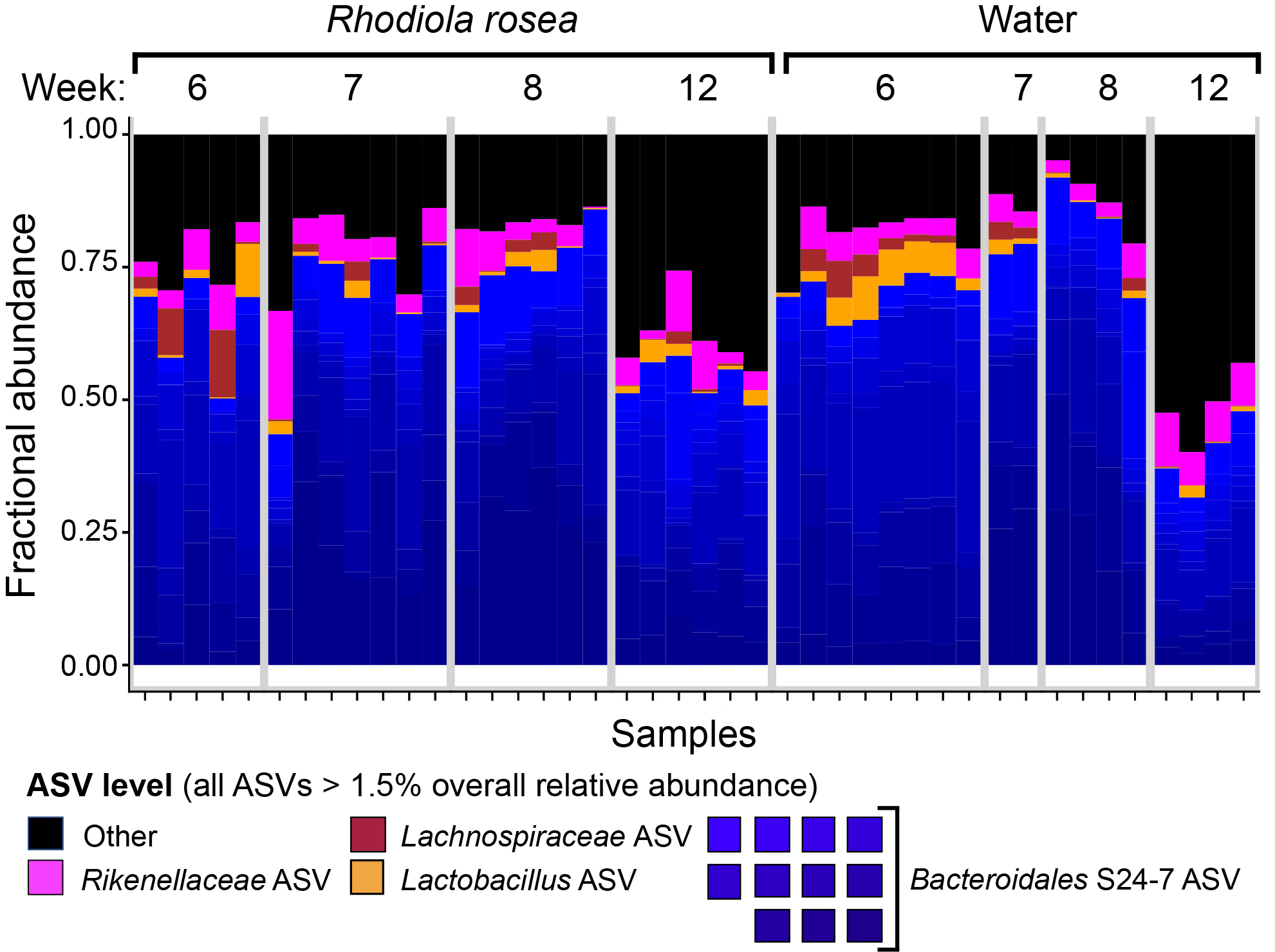


**Supplementary Fig. 1. Taxon plot of microbiome analysis.** Analysis of fecal bacterial abundance over the treatment period shows composition is dominated by bacteria from the *Bacteroidetes* phylum, especially from the *S24-7* family.


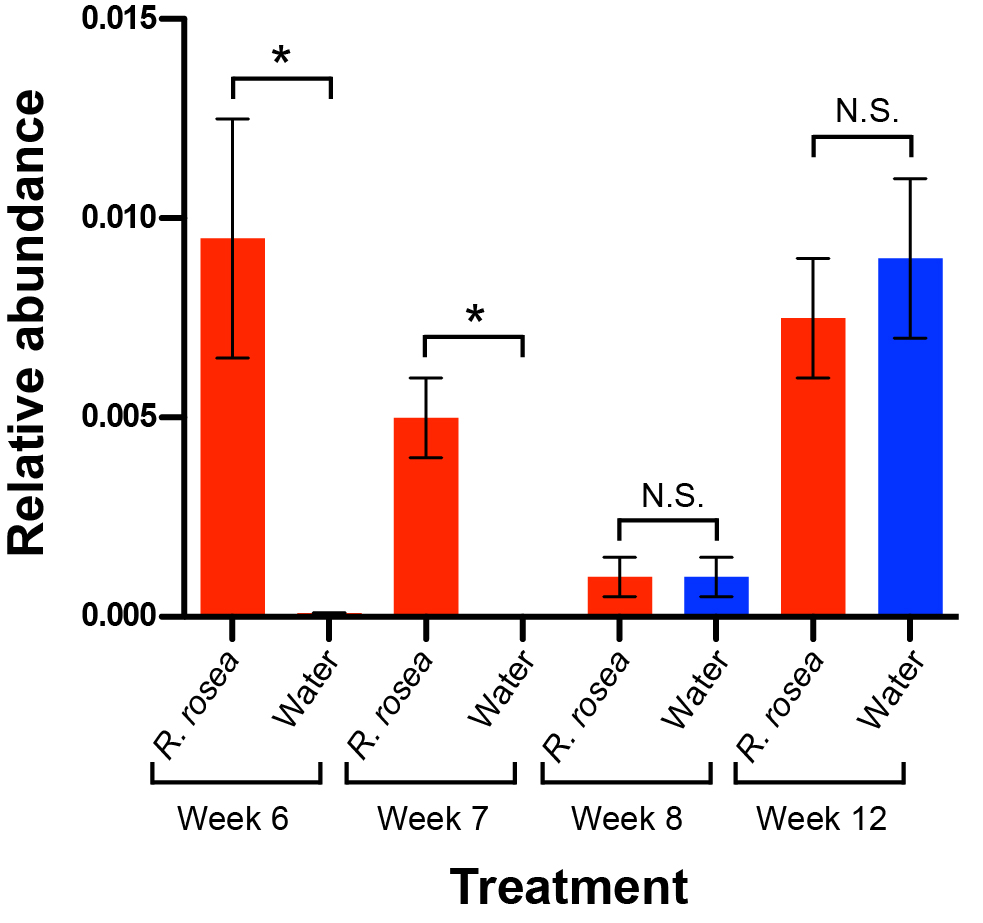


**Supplementary Fig. 2.** **Treatment differences in fractional abundance of bacteria from the *Desulfovibrionales* order at each timepoint sampled.** Higher abundances were observed in mice treated with *R. rosea* than the control at pretreatment (week 6) and one week following treatment (week 7). No statistically significant differences were observed at the week 8 and week 12. Statistically significant differences between relative abundance of the *Desulfovibrionales* at each time point were determined by ANCOM (**P*<0.05).


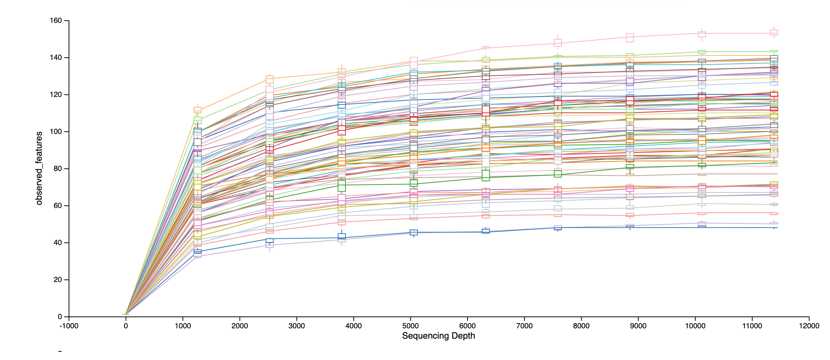


**Supplementary Fig. 3. Rarefaction curve of forward reads following quality filtering and denoising by default DADA2 parameters in QIIME2.** Forward reads were rarefied to 11,390 reads per sample, providing sufficient depth for pattern differentiation.

**Supplementary Table 1. Primers for Q-PCR.**

| **Gene** | **Sequence** |
| --- | --- |
| CRP_F | ATGGAGAAGCTACTCTGGTGC |
| CRP_R | ACACACAGTAAAGGTGTTCAGTG |
| GAPDH_F | AGGTCGGTGTGAACGGATTTG |
| GAPDH_R | TGTAGACCATGTAGTTGAGGTCA |
